# Interactions of Shiga-like toxin with human peripheral blood monocytes

**DOI:** 10.1007/s00467-007-0512-4

**Published:** 2007-08-01

**Authors:** Joyce M. Geelen, Thea J. A. M. van der Velden, Lambertus P. W. J. van den Heuvel, Leo A. H. Monnens

**Affiliations:** Department of Paediatric Nephrology, Radboud University Nijmegen Medical Centre, P.O. Box 9101, 6500 HB Nijmegen, The Netherlands

**Keywords:** Monocyte, Hemolytic uremic syndrome, Shiga-like toxin, Acute renal failure, HUVEC, vero cells

## Abstract

The cytotoxic effect of Shiga-like toxin (Stx; produced by certain *Escherichia coli* strains) plays a central role in typical hemolytic uremic syndrome (HUS). It damages the renal endothelium by inhibiting the cellular protein synthesis. Also, the monocyte has a specific receptor for Stx but is not sensitive for the cytotoxic effect. In this work, monocytes were studied as a potential transporter for Stx to the renal endothelium. Coincubation of isolated human monocytes loaded with Stx and target cells (vero cells and human umbilical vascular endothelial cells) were performed. Transfer was determined by measuring the protein synthesis of target cells and by flow cytometry. Furthermore, the effect of a temperature shift on loaded monocytes was investigated. Stx-loaded monocytes reduced the protein synthesis of target cells. After adding an antibody against Stx, incomplete recovery occurred. Also, adding only the supernatant of coincubation was followed by protein synthesis inhibition. Stx detached from its receptor on the monocyte after a change in temperature, and no release was detected without this temperature shift. Although the monocyte plays an important role in the pathogenesis of HUS, it has no role in the transfer of Stx.

## Introduction

Hemolytic uremic syndrome (HUS) is a clinical syndrome consisting of three characteristic features: hemolytic anemia, thrombocytopenia, and acute renal failure [1]. In the new classification of HUS, infections due to Shiga-like toxin (Stx)-producing bacteria belong to the category “etiologically advanced” [2]. This work focuses on the form in which Stx-producing *Escherichia coli* is the most common pathogen [1]. It can produce several types of Stx, of which Stx1, Stx2, and Stx2c are most frequently associated with HUS [3, 4]. Stx plays a crucial role in the pathogenesis because of its cytotoxic effect on the renal endothelium. Both renal tubular epithelial cells and glomerular visceral epithelial cells (podocytes) are also sensitive to the toxic effect of Stx [5, 6]. It can inhibit the protein synthesis of these cells after specifically damaging the ribosomal RNA [7]. However, the question of how this toxin is targeted mainly to the kidney remains unsolved. Stx was never detected in the serum of patients, but it was detected in renal biopsy material of patients with HUS [8]. As a specific treatment for HUS is still lacking, more insight into the transport of this toxin might lead to new intervention strategies.

After oral ingestion of the bacteria through contaminated food or water, the noninvasive bacteria adhere to the intestinal epithelial cells of the distal small bowel and colon. This leads to a rearrangement of the morphology of the cells and initiates inflammation [9, 10]. Bacterial flagellin plays an important role in this process [11]. Stx can probably reach the circulation because of active transport in these cells and also passively after damage to the intestinal cells [12]. Subsequently, it has to be transported in the circulation to reach its primary target, the renal endothelium.

It is very tempting to look at the blood cells as a carrier for the toxin. Stx can bind to a specific receptor, which is a globotriaosylceramide (Gb3, P^k^ Antigen, CD77) [13]. This receptor is present on renal endothelial cells but also on blood cells. Stx binding has been described on red blood cells [14], B lymphocytes [15], and platelets, which also have an additional binding possibility (glycolipid, band 0.03) [16]. Several groups showed the existence of a specific binding of Stx on monocytes [17, 18, 19]. After binding to its receptor, Stx can be internalized. Whereas in epithelial cells the toxin follows the retrograde transport route and becomes cytotoxic, in monocytes it is targeted to the lysosomes and will get degraded [19]. During this transport, the monocyte becomes activated. This will lead to an increase of transcription factors, such as nuclear factor kappa B (NF-κB) and activator protein 1 (AP-1), and an upregulated production of cytokines such as interleukin (IL)-1β, tumor necrosis factor (TNF)-α, IL-6, and IL-8 [17, 20]. These events will have a pro-inflammatory effect.

We postulated that, as the monocyte has a specific receptor, it might also function as a carrier to transport Stx to the renal endothelium. To investigate this hypothesis, Stx was loaded to isolated monocytes from healthy donors and coincubated with target cells [vero cells and human umbilical cord venous endothelial cells (HUVEC)]. The level of transfer was determined by measuring the protein synthesis of these target cells and the transfer of fluorescein isothiocyanate (FITC)-labeled B subunit of Stx1 with flow cytometry.

## Materials and methods

### Materials

Stx2 was kindly provided by Dr. M. Karmali (Public Health Agency of Canada, Ontario, Canada). FITC-labeled Stx1B subunit and ^125^I-Stx1B subunit were a gift from Dr. L. Johannes (Institut Curie, Paris, France). Stx1B subunit is a useful tool for studying binding in monocytes [19]. It is the binding part of the toxin, whereas the enzymatic A subunit will only stimulate the uptake of the toxin and does not affect binding [21]. Vero cell medium consists of M199 (Gibco; Paisley/UK), fetal calf serum (FCS, Greiner Bio-One; Kremsmunster/Austria), penicillin/streptomycin (Gibco, Paisley/UK), and glutamine (MP Biomedicals; Eschwege/Germany). HUVEC medium is made of M199, human serum (HS; Cambrex; Walkersville/USA), newborn calf serum (NBCS; Gibco, Paisley/UK), penicillin/streptomycin, glutamine, heparine (Leo Pharma BV, Breda/The Netherlands), and endothelial-cell growth factor [22].

Ethylenediamine tetraacetic acid (EDTA) tubes were purchased from BD Vacutainer (Alphen aan de Rijn/The Netherlands). The MACS kit for negative selection of monocytes was provided by Miltenyi Biotec (Bergisch Gladbach/Germany). Hank’s balanced salt solution (HBSS) was ordered from MP Biomedicals (Eschwege/Germany). Human serum albumin (HSA) from Sanquin (Amsterdam/The Netherlands) and porcine gelatin from Fluka (Neu-Ulm, Germany) was used. Trichloroacetic acid and TNF-α was obtained from Sigma-Aldrich Chemie B.V. (Zwijndrecht/The Netherlands). The antibody against Stx2 (TMA-15) is well characterised [23]. It was a kind gift from Dr. Yamagami from the Department of Biomedical Research from the Teijin Institute, Tokyo, Japan. ^3^H-leucine and Ficoll-paque PLUS was purchased from Amersham Biosciences (Uppsala, Sweden). Culture plates were ordered from Corning Inc. (Corning, USA).

### Culture of vero cells and HUVEC

Vero cells (renal epithelial cells of the African green monkey) were grown to confluency on 24-well plates (ordered from ATCC; Middlesex, UK). These cells have a high basal expression of Stx-receptor CD77. HUVEC were isolated, and these cells were grown to confluence on gelatin-coated 24-well plates [24]. Every 2 days, fresh medium was added to the cells. In contrast, HUVEC were preincubated for 24 h with TNF-α (10 ng/ml) to upregulate the expression of CD77.

### Isolation of monocytes and loading with Stx2

Fresh venous blood (20 ml) from 40 healthy donors was collected into EDTA tubes. Monocytes were isolated by negative selection using antibody-labeled beads (CD3, CD7, CD16, CD56, and CD123). After centrifugation of blood over Ficoll (20 min 400 g without break at room temperature), the interphase (containing monocytes, lymphocytes, and platelets) was collected. Platelets were removed by centrifugation (200 g 10 min at room temperature) before adding the beads. The purity of monocytes after the magnetic isolation was (as determined by flow cytometry) 80–85%. After the isolation, the monocytes were resuspended in HBSS with 1% HSA and placed on ice. In every experiment, monocytes from one donor were used. To load the monocytes with Stx2, the toxin was added to a concentration of 10 nM during a time period of 3 h [17]. The cells remained on ice. After proper washing to remove all unbound Stx2, the cells were resuspended in vero cell or HUVEC medium (in which HS and NBCS were substituted by FCS). Monocytes stayed viable after this loading, as determined by trypan blue exclusion.

### Coincubation Stx2-loaded monocytes and target cells

These experiments were performed in two different experimental settings: with transfer of loaded monocytes from 4° to 37°, and without change of temperature. To start with the first setting, the Stx2-loaded monocytes were added to a monolayer of target cells [HUVEC (*n* = 10) or vero- cells (*n* = 9)] in a concentration of 1 × 10^6^ per well. This was performed during 24 h at 37°C. For comparison, also monocytes without Stx were used. To determine the specificity of the effect of Stx2, the loaded monocytes were preincubated with a well-characterized antibody against Stx2 (TMA-15, 1 μg/ml, approximately 150× excess). All experiments were performed in duplicate. To measure the transfer of Stx2 from the monocyte, the protein synthesis of the target cells was determined by adding ^3^H-Leucine (0.67 μCi/ml). Subsequently, intracellular proteins were precipitated by treatment with trichloroacetic acid (TCA), and the radioactivity was measured in a liquid scintillation counter. In the other experimental setting, monocytes were loaded in a similar way with FITC-labeled Stx1B subunit; 1 × 10^6^-loaded monocytes were coincubated with 1.25 × 10^5^ vero cells in suspension for 3 h at 4°C (*n* = 5) while being continuously rotated. At this temperature, bias due to internalization of the toxin could be avoided [19, 25]. After 3 h, the presence of Stx1B subunit FITC on the vero cells was determined by flow cytometry. For every experiment, at least 1,000 cells were measured.

### Study of supernatant Stx-loaded monocytes

To study the effect of the supernatant of Stx2-loaded monocytes, it was collected after 16 h of coincubation on vero cells (*n* = 11). The supernatants were centrifuged to remove possible monocytes and added again to fresh vero cells. Protein synthesis was measured by adding ^3^H-leucine and measuring the incorporation after 24 h of incubation at 37°C. Also, the antibody against Stx2 was used to determine a possible effect of free Stx2. Furthermore, monocytes were loaded with ^125^I-Stx1B subunit to investigate whether it could be released after a change in temperature from 4° to 37°C (*n* = 3). For each experiment, between 3 and 4 × 10^6^ monocytes are loaded with 200 nM Stx1B subunit (3,600 cpm/ng protein). After loading, unbound Stx1B subunit was removed by centrifugation. Subsequently, the monocytes were placed at 37°C for 2 h. They were then centrifuged, and cells and supernatant were measured separately for the presence of ^125^I-Stx1B with a gamma counter. This was compared with the amount of binding to the monocytes before the temperature change. The experiment was performed in duplicate.

### Statistics

All data presented are expressed as a range with the median. Significance of increase or decrease of protein synthesis compared with controls was analyzed using the Wilcoxon signed ranks test. The statistical level of significance was defined as *P* < 0.05.

## Results

### Coincubation Stx2-loaded monocytes and target cells

**Fig. 1:**
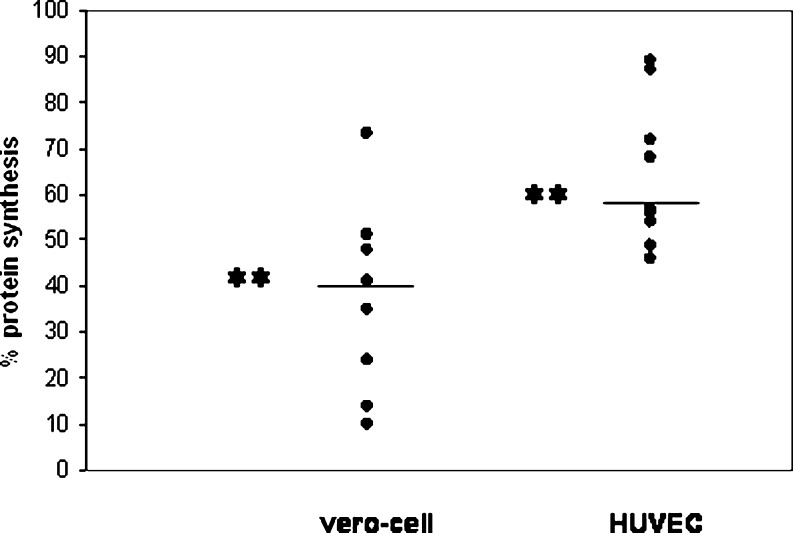
Coincubation of Shiga-like toxin (Stx)2-loaded monocytes with target cells [vero cells and human umbilical cord venous endothelial cells (HUVEC)]. To investigate whether monocytes could transfer Stx2, toxin-loaded monocytes were coincubated with vero cells (*n* = 9) and HUVEC (*n* = 10). This led to protein synthesis inhibition, as could be determined with the incorporation of ^3^H-leucine. The coincubation with unloaded monocytes was used as a control and set at 100%. ** *P* < 0.01

**Fig. 2:**
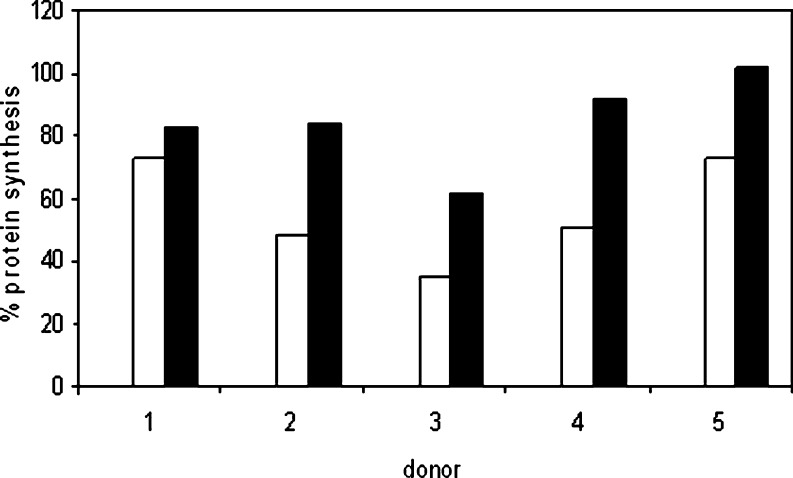
Effect of the addition of an antibody against Shiga-like toxin (Stx)2. The monocytes of five healthy donors were coincubated with vero cells (Stx2-loaded monocytes, *white bar*; Stx2-loaded monocytes with Stx2 antibody, *black bar*). Addition of the antibody against Stx2 led to partial protein synthesis recovery in four donors and complete recovery in one donor. Coincubation with unloaded monocytes was used as a control and set at 100%

To investigate whether monocytes can transfer Stx2 to target cells (vero cells and HUVECs), Stx2-loaded monocytes were added to a monolayer of these cells. After coincubation, protein synthesis of vero cells and HUVEC was measured. If the Stx2 was transferred to the target cells, there was protein synthesis inhibition, as this is the biological effect of Stx2 in both types of target cells. After 24 h of coincubation at 37°C, in both cell types, there was protein synthesis inhibition (Fig. 1). In vero cells, greater protein synthesis reduction was measured than in HUVEC. Directly adding 10 nM Stx2 to vero cells reduced protein synthesis to 5.5% (data not shown). When an antibody against Stx2 was added to the monocytes before coincubation with vero cells, protein synthesis inhibition was partly restored (Fig. 2). Only in one out of five experiments was there complete recovery.

In equal experiments performed with HUVEC (*n* = 3), TMA-15 also partly prevented protein synthesis inhibition (data not shown).

### Study of supernatant Stx-loaded monocytes

**Fig. 3:**
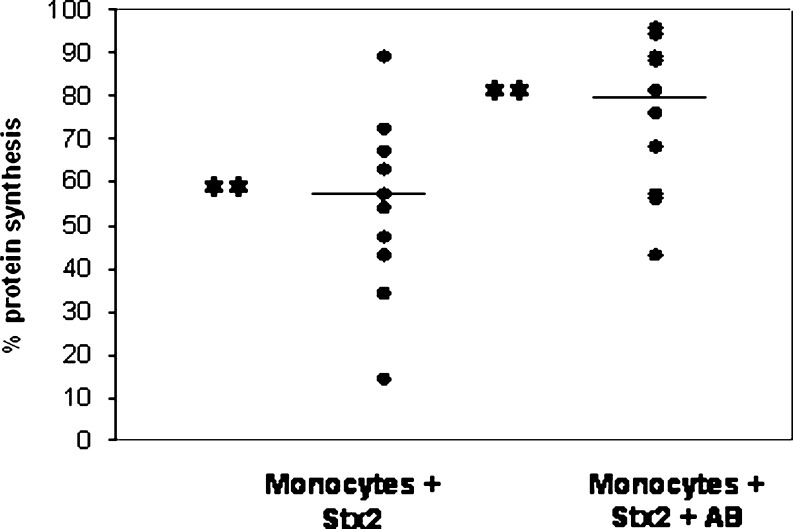
Addition of supernatants coincubation to vero cells. After coincubation of Shiga-like toxin (Stx)-loaded and unloaded monocytes, supernatants were collected and re-added to fresh vero cells (*n* = 11). The supernatant from the toxin-loaded monocytes induced, again, protein synthesis inhibition. This inhibition could be partially restored with a Stx2 antibody (AB). The incubation of supernatant with unloaded monocytes was used as a control and set at 100%. ** *P* < 0.01

Because the addition of an antibody against Stx2 was not sufficient for complete protein synthesis recovery in four out of five tested donors, we investigated the possible presence of an additional inhibitor in the supernatant after 16 h of coincubation. This was performed by re-adding the supernatant of coincubated Stx2-loaded monocytes and a monolayer of vero cells to new vero cells. Figure 3 shows that this incubation again led to an inhibitory effect on vero-cell protein synthesis in contrast to the supernatant of unloaded monocytes. But this reduction was less than with direct coincubation. The effect could be partly blocked by adding the antibody against Stx2. This means that there is unbound Stx2 present in the supernatant. Next, we investigated whether the temperature change of the Stx-loaded monocytes from 4° to 37°C could lead to toxin release. For this reason, we loaded the monocytes with ^125^I-Stx1B subunit. The amount of radioactivity on or inside the monocytes was measured before and after incubation of the cells for 2 h at 37°C (Table 1). Vero cells were used as a control. In vero cells as in monocytes, the toxin was released from its receptor after the incubation.

**Table 1:** Amount of ^125^I- Shiga-like toxin (Stx)1B subunit on cells after change of temperature

Stx-loaded cells	Before 4→37°C (cpm)	After 4→37°C (cpm)
Vero cells	1,034,383	671,060
Monocytes donor 1	1,437	427
Monocytes donor 2	1,070	374
Monocytes donor 3	1,121	677

*cpm* counts per minute

### Transfer of Stx to target cells without change in temperature

**Fig. 4:**
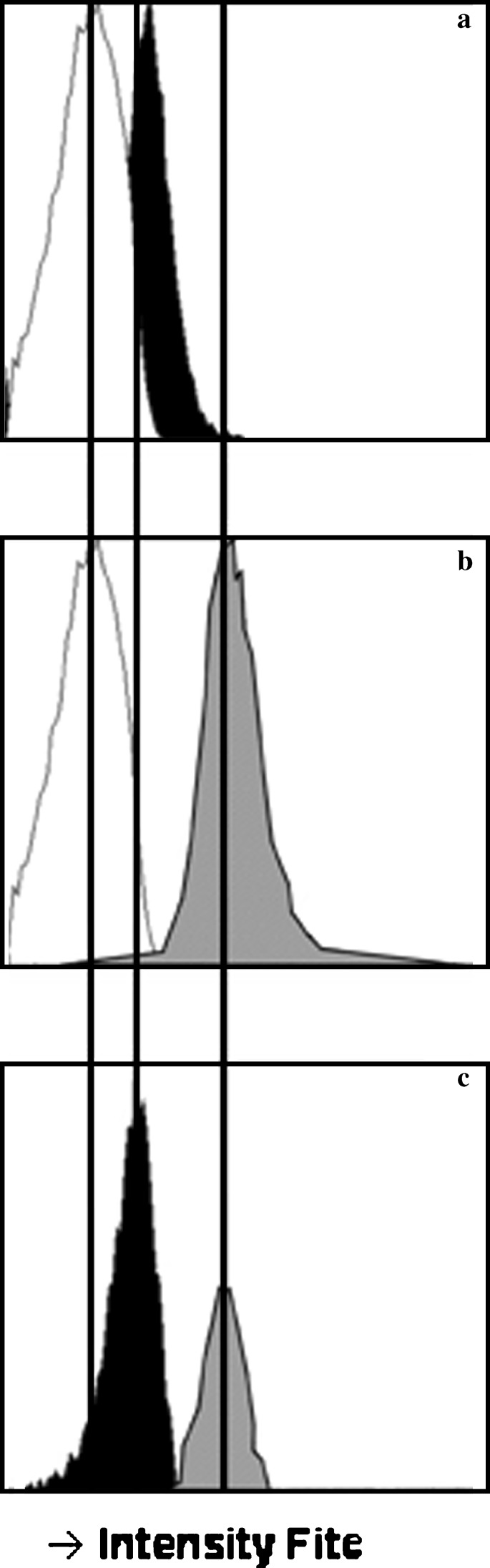
Transfer of Shiga-like toxin (Stx)-loaded monocytes to vero cells without temperature shift (*white*: normal monocytes; *black*: Stx-loaded monocytes; *grey*: vero cells). The *x-axis* represents the intensity of the fluorescein isothiocyanate (FITC) signal, the *y-axis* represents the number of cells. **a** FITC-labeled Stx1B subunit was bound to monocytes from a healthy donor, showing an increase in intensity. **b** Coincubation of vero cells and unloaded monocytes; note the high basal signal of vero cells. **c** After 2 h of coincubation with vero cells in suspension at 4°C, there was no transfer of the FITC signal. The cells remained in position

A change in temperature from 4° to 37°C released Stx from its receptor on the monocyte. To study again the possibility of Stx transfer from monocyte to target cells, we loaded isolated monocytes with Stx1B subunit labeled with FITC. Coincubation of these monocytes with vero cells in suspension without a change in temperature did not result in a transfer of the B subunit to the vero cells (*n* = 5, Fig. 4).

## Discussion

**Fig. 5:**
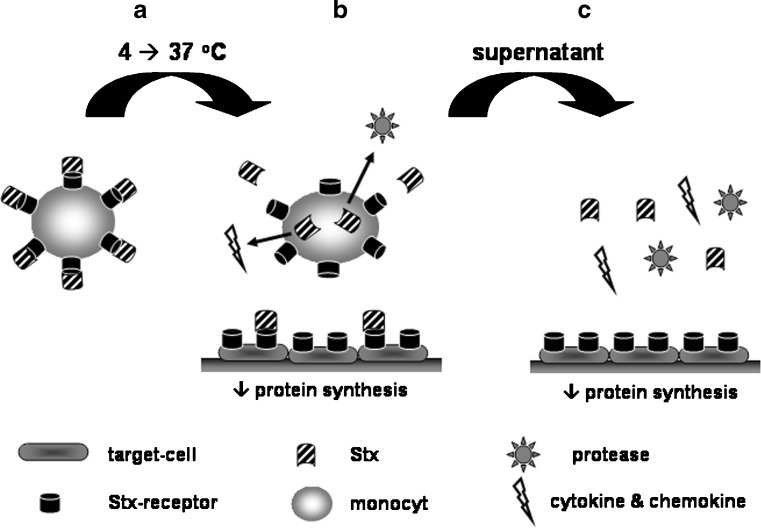
Schematic summary of performed experiments. **a** Monocytes loaded with Shiga-like toxins (Stx) at 4°C. **b** After shifting Stx-loaded monoytes to target cells at 37°C, Stx was released from its receptor. This led to protein synthesis inhibition of target cells. However, transport could not be excluded (but this was performed in experiments without a shift in temperature). Probably, some toxin was internalized and monocytes became activated. This could lead to cytokine and proteases production (possible cytotoxic factors). **c** When the supernatant is re-added to new target cells, there are released Stx and secreted products present. This induced, again, protein synthesis inhibition

After coincubation of Stx2-loaded monocytes with target-cells (vero cells and HUVEC), protein synthesis inhibition could be detected. As the biological effect of Stx is protein synthesis inhibition, a transfer was expected. This effect could not be due to the presence of monocytes, because it is well described that Stx has no inhibitory effect on peripheral blood monocytes [17]. However, the addition of an antibody against Stx only partly restored the inhibition. We hypothesized that in parallel to transfer of Stx2, a possible additional inhibiting factor was present in the supernatant during the experiment. To further investigate this possibility, the supernatant of this coincubation was re-added to fresh vero cells. Again, there was protein synthesis inhibition, which could partly be decreased by blocking with the Stx2 antibody. The conclusion was made that there another inhibitory factor needed to be present (possibly released by activated monocytes), but unbound Stx2 was also present [26]. Apparently, Stx1B subunit is released from its receptor when there is shift of temperature from 4° to 37°C, as shown in this study and also by Ramegowda and Tesh [18]. This finding stresses the caution that must be taken when drawing conclusions from in vitro experiments performed at 4°C. In Fig. 5, all findings are schematically summarized.

As these in vitro experiments were not suitable to investigate a possible transfer, experiments were performed without a change of temperature. No transfer of the binding part of the toxin could be detected in this setting. Experiments could not be performed at 37°C because the toxin is internalized by the monocytes during 3 h of incubation. Because of these in vitro experiments, we believe that the monocyte cannot function as a transporter for Stx in the circulation. However, monocytes still seem to play an important role in pathogenesis. As a component of the innate immune system, they play a central role in immunity and inflammation. Fernandez et al. showed that patients in the acute period of HUS have monocytes with phenotypic (reduced expression of CD14, CD64, and CD11b) and functional [decreases lipopolysaccharide (LPS)-induced TNF-α production and Fcγ-dependent cytotoxicity] differences compared with healthy children and acute uremic children [27].

Some unanswered questions remain. What is the inhibitory factor released by monocytes after loading with Stx? Why is there still unbound Stx2 present in the supernatant after 16 h of coincubation? It is surprising that Stx2 is not completely bound and internalized by the numerous receptors on monocytes and vero cells. Also, how Stx is transported in the circulation remains unsolved. Kimura et al. described that serum amyloid P (SAP) can bind Stx2 and function as a neutralizing factor [28]. However, in humans, there was no correlation between circulating SAP and the development of HUS [29].

As renal endothelial damage is already present after the occurrence of clinical symptoms of HUS, it is very important to develop efficacious early prevention. Understanding the mechanism in which the toxin is specifically targeted to the kidney can lead to novel intervention strategies.
